# Olden’s Contributions

**DOI:** 10.1289/ehp.112-a866b

**Published:** 2004-11

**Authors:** Rudi H. Nussbaum

**Affiliations:** Professor Emeritus, Physics and Environmental Sciences, Portland State University, Portland, Oregon, E-mail: D4RN@odin.pdx.edu

I read with mixed feelings of approval and sadness your editorial about the end of tenure for Kenneth Olden as director of the National Institute of Environmental Health Sciences (NIEHS) (Brown et al. 2004). I have been greatly impressed with Olden’s significant contributions to broadening the scope of public health sciences, as reflected in the evolution of *EHP* into an exemplary, innovative, and internationally highly respected journal on environmental health sciences.

Specifically, the following sentence in the editorial was grist for my mill:

From early on he showed awareness and understanding of a fact that had often been ignored by others in research administration—that local communities have the collective ability to identify environmental health problems but often lack the time, means, and research expertise to effectively resolve these problems.

We have just published a report describing a unique community–physician–scientist cooperative research effort without support from any public agency that has been—at least for a small number of survivors of this group of Hanford, Washington, “downwinders”—of great significance for their experiencing a sense of empowerment and, at least to some degree, of “justice” through the process of scientific validation (Nussbaum et al. 2004).

It seems that the efforts of our alliance might well have fallen within the boundaries of projects that Olden’s initiatives could have supported: to provide and link communities with appropriate research resources.

I fear that Olden’s departure will be a great loss for the NIEHS and that it will be very difficult to find a replacement for him with an equally bold vision and willingness to take risks in innovative leadership.
